# Social preferences for health states associated with acute myeloid leukemia for patients undergoing treatment in the United Kingdom

**DOI:** 10.1186/s12955-018-0897-8

**Published:** 2018-04-18

**Authors:** Nacho Castejón, Joseph C. Cappelleri, Jesús Cuervo, Kathryn Lang, Priyanka Mehta, Ruth Mokgokong, Carla Mamolo

**Affiliations:** 1 HEOR Senior Consultant, Barrio de Abajo, “Las Barcas”, 33595 Celorio, Spain; 2 Pfizer Inc, 445 Eastern Point Road, MS 8260-2502, Groton, CT 06340 USA; 3 LA-SER Research España, C/Condado de Treviño 2, Portal 1-Bajo 3, 28033 Madrid, Spain; 4 Pfizer Ltd, Walton Oaks, Dorking Road, Tadworth, Surrey, KT20 7NS UK; 5 Bristol Haematology Oncology Centre, University Hospitals Bristol NHS Trust, Horfield Road, Bristol, BS2 8ED UK

**Keywords:** Acute myeloid leukemia, Health-related quality of life, Time trade-off, Health states, Utility values

## Abstract

**Background:**

Health state (HS) utility values for patients with acute myeloid leukemia (AML), a hematological malignancy, are not available in the United Kingdom (UK). This study aims to develop clinically sound HSs for previously untreated patients with AML and to assign utility values based on preferences of the general UK population.

**Methods:**

This study was conducted in the UK and comprised 2 stages. During the first stage, AML HSs were drafted based on evidence from a literature review of AML clinical and health-related quality-of-life studies (published January 2000–June 2016) and patient-reported outcome measures previously used in this population. A panel of UK hematologists with AML experience validated the clinical relevance and accuracy of the HSs. During the second stage, validated HSs were valued in an elicitation survey with a representative UK population sample using the time trade-off (TTO) method. Descriptive statistics and bivariate tests were obtained and performed.

**Results:**

A total of eight HSs were developed and clinically validated, including treatment with chemotherapy, consolidation therapy, transplant, graft-vs-host disease (GvHD), remission, relapse, refractory, and functionally cured. In total, 125 adults participated (mean age, 49.6 years [range, 18–87 years], 52.8% female). Mean (95% confidence interval [CI]) TTO preference values (*n* = 120), ranked from lowest (worst HS) to highest (best HS) were as follows: refractory − 0.11 (− 0.21 to − 0.01), relapse 0.10 (0.00–0.20), transplant 0.28 (0.20–0.37), treatment with chemotherapy 0.36 (0.28–0.43), GvHD 0.43 (0.36–0.50), consolidation 0.46 (0.40–0.53), remission 0.62 (0.57–0.67), and functionally cured 0.76 (0.72–0.79). Mean (95% CI) visual analog scale preference values followed the same rank order, ranging from 0.15 (0.13–0.17) for refractory to 0.71 (0.68–0.73) for functionally cured.

**Conclusions:**

To our knowledge, this is the first study to report utility values for AML from the UK societal perspective. Participants were able to distinguish differences in severity among AML HSs, and preference values were consistent with clinical perception of HS severity. HS preference values observed in this study may be useful in future evaluations of treatment benefit, including cost-effectiveness analyses and improved patient well-being.

## Background

Acute myeloid leukemia (AML), a hematological malignancy, represents approximately one-third of all leukemia cases in the United Kingdom (UK) [1]. In 2014, approximately 3072 individuals were diagnosed with AML in the UK [1]. The incidence is slightly higher in males (57%) than females (43%), [1] and increases with age [1]. Between 2012 and 2014, more than half (approximately 55%) of AML cases occurred in individuals ≥70 years old, with the peak rate in individuals ≥85 years old [1]. Although remission rates with chemotherapy are high (approximately 80%) in newly diagnosed patients, overall survival is low (5 to 6 year OS, approximately 40%) and decreases in older patients [2, 3].

The treatment and management of patients with AML comprises front-line induction therapy and consolidation/maintenance therapy [4, 5]. In addition to chemotherapy, select patients will receive hematopoietic stem cell transplantation as part of their therapy because it is associated with superior treatment outcomes [6, 7]. Most often, induction and consolidation regimens utilize intensive chemotherapy [4]; however, treatment approaches may vary depending on whether the intent of treatment is curative, prolonging survival, or palliative.

A combination of patient- and disease-related factors, such as age, comorbidities, performance status, and prognostic cytogenetic/genetic markers, also influences treatment decisions [4]. In general, complete remission rates, median overall survival, and median disease-free survival are lower in older patients compared with younger patients [8–11]. Additionally, in patients with poor performance status, the likelihood of dying early after remission induction increases substantially with increasing age [8]. Thus, treatment goals are based on a comprehensive assessment of patient- and disease-related prognostic factors and whether patients are likely to tolerate therapy.

Throughout the course of their disease, patients with AML experience adverse events and toxicity as a result of treatment. Distressing symptoms are common in patients receiving induction therapy, with approximately half of patients experiencing severe levels of distress early on after diagnosis [12]. Health-related quality of life (HRQoL) is significantly lower in patients with AML compared with the general population [13], and newly diagnosed patients experience greater reductions in HRQoL compared with patients with longer times since diagnosis [14]. Improvement in HRQoL has been observed over time; however, stage of disease (eg, remission vs terminal disease) and specific treatments (eg, allogeneic stem cell transplant) negatively affect HRQoL [13–15].

Health-related quality of life outcomes, along with health-economic evaluations, are an important component of assessing the efficiency of treatment interventions. In the UK, guidance on health technology appraisal set forth by the National Institute for Health and Care Excellence (NICE) expressly stipulates that economic evaluation (specifically cost-utility analysis) is a component of health technology appraisal [16]. Quality-adjusted life-year (QALY), a value-weighted measure of health improvement, is the recommended outcome measure for cost-utility analysis [16, 17]. QALY is the product of life expectancy and the utility value associated with a particular condition of a patient in various situations that occur as a result of disease- or treatment-related factors (known as a health state [HS]) [16–18].

Utility values measure and assign valuation to an individual’s preference for a particular HS [17, 18]. Utility values typically range from 0 (death) to 1 (full health), and states worse than death are expressed as negative values [17]. Utility scores can be derived from generic HRQoL measures, such as the EuroQol 5-Dimension Questionnaire (EQ-5D) [19], preferred by NICE, or obtained from other appropriate published studies reporting EQ-5D data [16]. NICE also prefers that HRQoL changes be obtained directly from patients, but that the values of the derived HSs are based on public preferences (to better reflect social desirability of the HS). In the absence of EQ-5D data, alternative measures or estimates of health benefit can be utilized, if available [16]. Methods to measure utility include preference elicitation studies, using methods such as standard gamble, Visual Analog scale (VAS), and time trade-off (TTO; preferred by NICE) [16–18]. Utility values and their use in cost-effectiveness analysis inform many decisions in health care resource utilization and are beneficial in understanding HSs associated with a particular disease.

Health state utility values for patients with AML are not available in the literature to our knowledge, neither derived from preferred measures (ie, EQ-5D), nor directly elicited from HS descriptions. Therefore, it is important to identify HS utility values applicable to patients with AML. This is the first study to define and clinically validate HSs experienced by AML patients and to estimate the associated utility values for each of these HSs from the perspective of the general UK population.

## Methods

### Study design

This non-interventional, cross-sectional study conducted in the UK between April 2016 and October 2016 was performed in 2 stages. In Stage 1, AML HSs were developed and validated. In Stage 2, HS valuation was achieved using a sample representative of the UK population.

Eligible participants were ≥ 18 years old, resided in the UK, and provided written informed consent. Participants with cognitive impairment, hearing difficulty, visual impairment, or of altered mental state (eg, under the influence of drugs or alcohol), as judged by the interviewer, were excluded. Participants unwilling to provide written informed consent were also excluded.

### Health state development and validation

Health states were developed to include information about the treatments that participants are receiving, the symptoms they might be experiencing, the impact on their daily lives, and the outlook for the future. Descriptions were based on evidence from a literature review of AML clinical and HRQoL studies (published January 2000–June 2016). A search of Medline, Cochrane Database of Systematic Reviews, Cochrane Central database, Database of Abstracts of Reviews of Effects, Technology Assessment Database, and International Prospective Register of Systematic Reviews (National Institute for Health Research Centre for Research and Dissemination) was performed to identify existing utility studies and any relevant HSs in AML.

Additional sources, including the National Health Services Economic Evaluation Database, Tufts Medical Center Cost-Effectiveness Analysis Registry, Value in Health, NICE [20] and Clinicaltrials.org, were searched to identify utility values related to AML. Search terms included acute myeloid leukemia, quality of life, health-related quality of life, utility, cost-utility, azacitidine, induction therapy, treatment, longitudinal, long-term, symptoms, chemotherapy, stem cell transplant, bone marrow transplantation, transfusion, side effects, recurrence, relapse, graft-versus-host disease, survivor, end of life, and supportive care. Published studies were also reviewed for relevant references.

A survey of the literature did not identify any utility studies in AML; therefore, the HSs were developed based on information gathered from the literature review, including clinical descriptions of the disease and patient-reported outcome measures used previously in patients with AML (eg, the European Organization for Research and Treatment of Cancer QLQ-C30 [21] and Functional Assessment of Cancer Therapy – Leukemia [22]).

A panel of UK hematologists with AML experience validated the clinical relevance and accuracy of the HSs. Based on feedback, amendments were made to include symptoms such as hair loss and psychological issues, clarify terminology, and better describe the outlook for patients.

### Health state valuation

Part 1 of HS valuation was a pilot phase tested in 10 participants. All procedures and materials to be used in part 2 were tested to identify practical problems with data collection, participant understanding of the procedures and the HS descriptions used, and validating HS descriptions. To reduce possible sources of bias, debriefing sessions with interviewers were held to ensure a systematic approach for conducting the interview was taken. Part 2, the main data collection phase, utilized VAS and TTO methodologies to valuate HSs. TTO preference values were the primary outcome, while the VAS was intended to introduce participants to the study because of its simplicity and easy-to-understand methodology, serving also as a check for coherence on the TTO responses.

### Data collection

Data collection and coding was obtained through 1:1 interviews completed by trained interviewers. Participants were identified by a third-party vendor and contacted by telephone, email, or direct mailing to ensure equal opportunity to participate and were selected by cluster random sampling and simple random sampling techniques. Cluster random sampling was applied based on UK regions/postcodes. For simple random sampling, participants were randomly selected from each region/postcode while monitoring for key sociodemographic variables, including age, gender, education, and employment status.

Interviews were conducted face-to-face (between August–September 2016) in single visits at either a central location in major cities across the UK or by visiting respondents at their home at an agreed date and time. Questionnaires were administered by the interviewer using Computer Assisted Personal Interviewing methodology. A total of 3 interviewers with experience in conducting face-to-face interviews collected all data and were given appropriate training for this study.

All data was captured in real time and stored on a secure server system, and all participants were assigned a numerical code. The interview included 3 sections: (1) sociodemographic data, (2) self-assessment of health, and (3) valuation exercise on the HS. After completion of all interviews, the interviewers recorded their impressions of the interview in a brief quality assessment that included questions related to degree of cooperation from the respondent, the respondent’s difficulty answering questions, and overall impression/quality of the response. A mean global interview score was calculated based on the sum of responses to the 3 questions comprising the interviewer’s assessment (degree of cooperation [potential score, 1−3]; trouble answering questions [1−5]; and global impression of the interview [1−5]). The range of possible scores was 3−13, with a higher score equivalent to a better experience. The interviewers’ assessments were used in cases concerning the quality of responses from participants or if a participant was not engaged or struggled to provide responses.

### Ethical approval

Using the online Health Research Authority Decision tool, it was determined that UK ethical approval was not needed for this study because it was considered to be minimal risk and was non-interventional, with participants answering questions in an interview setting [23]. However, the study design and materials were reviewed and approved by Salus IRB [24], a US-based institutional review board (IRB) accredited by the Association for the Accreditation of Human Research Protection Programs. All participants gave their written informed consent to participate in the study.

### Statistical analysis

Visual Analog Scale preference values were scored directly using the VAS for each possible HS; scores ranged from 0 to 100. VAS preference values were rescaled to a 0–1 range so they could be compared with results provided by the TTO method.

Time trade-off preference values were derived from the interview using the TTO method (1 value per HS). Each HS was categorized as better than death (BTD) or worse than death (WTD), and preference values ranged from −1 to 1, with 0 considered to be equal to death. For HSs BTD, the TTO preference value will be calculated as X/*t* where X is the time in full health that the participant is ready to trade for *t* time in the HS. For HSs WTD, the TTO preference value will be calculated as −X/*t* where X is the time in full health that the participant needs to stay in, so that X years in full health and (*t*−X) years in the HS are preferable to death for the participant.

Sample size was determined based on previously conducted similar studies and was to be representative of the UK population. Including the 10 participants from the pilot phase, the number of participants needed to estimate the mean utility value attached to a particular HS with a margin of error < 10% and to account for up to 15% of participants not being able to successfully complete the questions, or those that would provide inconsistent answers, was determined to be 125 adults.

Correlation of TTO and VAS preferences was completed using the Spearman rank correlation. Two-sample *t* tests were used to compare differences in mean preference values between female versus male participants. Spearman’s rank correlation was used to assess the correlation between the participant’s age and preference values.

## Results

### Health state development and validation

A total of 8 HSs were developed, validated by expert hematologists, and tested in the pilot study. Background information describing the disease and relevant symptoms was also developed (Table 1). The 8 HSs were as follows: treatment with chemotherapy (describes a patient undergoing treatment in the hospital, either induction or salvage therapies), consolidation (describes a patient undergoing consolidation therapy), transplant (describes a patient just after receiving a bone marrow transplant), graft-versus-host disease (GvHD; describes a patient who experiences GvHD after transplant), remission (describes a patient in remission), relapse (describes a patient that has been in complete remission and is now having a relapse and receiving best supportive care), refractory (describes a patient that has been in treatment and is not responding to it), and functionally cured (describes a functionally cured patient).

**Table 1:** Health States

Background	You have been diagnosed with a life-threatening disease that if left untreated will result in death. Your disease predominantly affects the blood, but there are many associated side effects that may be experienced as a result of this condition and its available therapies. These include hair loss, tiredness, weakness, weight loss, infections, psychological issues such as depression, anxiety and impairment of physical and sexual relations, fever, and fertility. Your ability to work and carry out normal daily activities will be disrupted. You are worried about the future with regards to issues such as treatment success, financial issues, and family relations. You don’t know who to tell, and what to tell them.
Treatment with chemotherapy	
Describes a patient undergoing treatment in the hospital, either induction or salvage therapies	You are receiving a treatment that requires you to stay alone in a hospital room for at least a month. You are at high risk of infection so visitors are limited. You require a blood transfusion every 2−3 days. You are being administered antibiotics, and treatment is given via a drip. You are subject to frequent blood tests and scans. You experience symptoms such as fatigue, diarrhea, nausea, pain, weight changes, and eating difficulties. Your family and social life have been disrupted, and you are unable to work. You feel nervous about the treatment outcomes.
Consolidation	
Describes a patient undergoing consolidation therapy	Your doctor has told you that the treatment has worked well so far. However, you now need more treatment to keep the disease from returning, requiring you to be at the hospital again for about 3 weeks, receiving blood transfusions every 2−3 days and treatment through a drip. When you’re no longer staying in the hospital, you do have to go back about twice a month for treatment and tests; and when you’re at home, you’re largely confined to the house. You still feel quite tired and experience symptoms during treatment, including fatigue, diarrhea, nausea, pain, weight changes, and eating difficulties. You are not able to perform your usual activities and are unable to work. You feel physically better, but are very worried about the disease coming back, or requiring a bone marrow transplant, both of which your doctor says are possibilities.
Transplant	
Describes a patient just after receiving a bone marrow transplant	The treatment has worked so far, and you are now receiving a bone marrow transplant as the next stage of your treatment, which is meant to ensure that the disease does not return. You need to be hospitalized for more than a month in an isolation room. You are on a drip for most of the time and receive blood transfusions every 2−3 days. The side effects of the treatment are much worse than you imagined: you are incapacitated by symptoms such as pain, diarrhea, nausea, dry mouth, and feel very fatigued. At times you need assistance with eating and have to be fed through a tube. You are not able to perform your usual activities and are unable to work. You are very worried as the doctors have told you that you may die from the transplant; you feel nervous but are hopeful for the future.
GvHD	
Describes a patient who experiences GvHD after transplant	You have now undergone a bone marrow transplant. This has been successful in its aim of reducing your disease; however, you have undergone a complication with this treatment. This has caused you to experience some additional side effects such as skin itchiness, diarrhea, poor mobility, and loss of appetite. As a result, you have been prescribed more medication and have to return to the hospital twice a week for blood tests and appointments. Your risk of infection is high; thus, contact with family and friends is limited. This also means that the time you spend outside the home must be reduced. Currently, you are allowed to be at home, but there is the chance of hospitalization if your condition worsens.
Remission	
Describes a patient in remission	Your treatment has now finished and you have been told that it has worked, but your doctor continues to monitor you every month and has warned you that there is still a risk that the disease could return and you’ll need further treatment. You have suffered a lot of side effects from which you have still not fully recovered: you are weak, very tired, and have a poor appetite. You start to resume your social life and usual activities, although in a very limited manner. You are relieved because the treatment worked but you still worry a lot about the disease coming back.
Relapse	
Describes a patient that has been in complete remission and is now having a relapse. The patient is receiving best supportive care	You have responded to treatment so far but your disease has now returned. Your doctor has advised you that at this point, curing your disease is unlikely if not impossible. You are at home but unable to perform your usual daily activities. A routine is difficult to maintain as you have to return to the hospital every 2 weeks to receive a blood transfusion until your future care is decided. Some of the symptoms of your disease are returning, including fatigue, loss of appetite, and nausea. You are still at high risk of infection; thus, contact with family and friends is limited, along with the time that you are allowed to spend outside of your home. In this situation, you feel depressed and anxious. You are worried about the future and whether there is any treatment left available for your condition. You are considering end of life care.
Refractory	
Describes a patient that has been in treatment and is not responding to it	Despite many treatments and suffering from their severe side effects, none of them have worked. The disease is no better, and you have been told you have no further treatment options. You are at home but unable to perform your usual daily activities. A routine is difficult to maintain as you have to return to the hospital every 2 weeks to receive a blood transfusion. Some of the symptoms of your disease are returning, including fatigue, loss of appetite, and nausea. You are still at high risk of infection; thus, contact with family and friends is limited, along with the time that you are allowed to spend outside of your home. In this situation, you feel depressed and anxious. You are worried about the future, and considering end of life care.
Functionally cured	
Describes a functionally cured patient	Your treatment has worked, but your doctor continues to monitor you. You think your overall health is good and you’re physically able to perform most of your usual activities, although you may have trouble with physical relationships. You are relieved because the treatment worked, and you plan to go back to work in the near future. You still sometimes worry about the disease coming back.

*GvHD* graft-versus-host disease

### Participants

A total of 125 adults participated in the study (pilot test phase, *n* = 10; main data collection phase, *n* = 115). Mean (range) age was 49.63 (18–87) years old, and 52.8% were women (Table 2). Assessment of health history demonstrated that 12.8% of participants reported a lifetime hematological disorder.

**Table 2:** Sociodemographics and Health History Information

Characteristic^a^	Participants (Interviewees) (*n* = 125)
Age, mean (SD), y	49.63 (16.16)
Range	18–87
Female	66 (52.8)
Educational level	
A Levels 1–3 (below University)	24 (19.2)
Levels 4–6 (University+)	101 (80.8)
Self-perceived health: mean VAS score (SD)	76.61 (14.73)
Suffering a chronic disease	54 (43.2)
Suffering an acute disease	10 (8)
Lifetime hematological disorder	16 (12.8)
Lifetime hematological disorder in close friends/relatives	21 (16.8)
Geographic areas	
East Midlands	7 (5.6)
East of England	11 (8.8)
London	19 (15.2)
North East	5 (4.0)
North West	15 (12)
Northern Ireland	4 (3.2)
Scotland	10 (8.0)
South East	17 (13.6)
South West	9 (7.2)
Wales	6 (4.8)
West Midlands	12 (9.6)
Yorkshire and the Humber	10 (8.0)

*SD* standard deviation, *VAS* Visual Analog scale

No participants had missing information for any characteristic

^a^Data are n (%) unless otherwise indicated

### Interviewer’s subjective assessment of interviews

Overall, interviewers rated all participants (ie, interviewees) as co-operative. The global impression of the interview was very good (58.4%) or good (39.2%) in most participants, and few participants (2.4%) had a great deal of trouble completing the exercises. The mean (SD) global interview score was 9.01 (0.79), indicating a high level of interview quality as determined by the interviewers.

### Preference values

A total of 5 participants did not engage in the TTO exercise because they were not willing to trade off any life-years. These participants were excluded from the primary analysis of TTO preference values (main analysis population, *n* = 120). HSs with the lowest TTO preference values, indicating poorer HS, were refractory, relapse, and transplant (Table 3). Higher preference values were observed for treatment with chemotherapy, GvHD, and consolidation, with the highest preference values (best HSs) observed for remission and functionally cured.

**Table 3:** Time Trade-Off Preference Values

Health State	Main Analysis Population (*n* = 120)	Alternate Analysis Population (*n* = 116)	All Participants (*n* = 125)
HS-1 Treatment with chemotherapy			
Mean (95% CI)	0.36 (0.28–0.43)	0.35 (0.27–0.42)	0.37 (0.30–0.45)
Median	0.50	0.50	0.50
Q1–Q3	0–0.60	0–0.60	0–0.60
HS-2 Consolidation			
Mean (95% CI)	0.46 (0.40–0.53)	0.46 (0.39–0.52)	0.47 (0.41–0.53)
Median	0.50	0.50	0.50
Q1–Q3	0.20–0.70	0.20–0.70	0.20–0.70
HS-3 Transplant			
Mean (95% CI)	0.28 (0.20–0.37)	0.27 (0.18–0.36)	0.30 (0.22–0.38)
Median	0.50	0.50	0.50
Q1–Q3	0–0.60	0–0.60	0–0.60
HS-4 GvHD			
Mean (95% CI)	0.43 (0.36–0.50)	0.42 (0.35–0.50)	0.44 (0.37–0.51)
Median	0.50	0.50	0.50
Q1–Q3	0.20–0.70	0.20–0.70	0.20–0.70
HS-5 Remission			
Mean (95% CI)	0.62 (0.57–0.67)	0.62 (0.57–0.67)	0.62 (0.57–0.67)
Median	0.70	0.70	0.70
Q1–Q3	0.50–0.90	0.50–0.83	0.50–0.90
HS-6 Relapse			
Mean (95% CI)	0.10 (0–0.20)	0.09 (−0.01–0.19)	0.12 (0.03–0.22)
Median	0.10	0.10	0.10
Q1–Q3	−0.10–0.50	− 0.10–0.50	− 0.10–0.50
HS-7 Refractory			
Mean (95% CI)	−0.11 (− 0.21 to − 0.01)	−0.11 (− 0.22 to − 0.01)	−0.08 (− 0.18–0.02)
Median	− 0.10	−0.10	− 0.10
Q1–Q3	−0.60–0.30	− 0.63–0.30	−0.50–0.50
HS-8 Functionally cured			
Mean (95% CI)	0.76 (0.72–0.79)	0.76 (0.72–0.80)	0.75 (0.71–0.79)
Median	0.80	0.80	0.80
Q1–Q3	0.70–0.90	0.70–0.90	0.70–0.90

*CI* confidence interval, *GvHD* graft-versus-host disease, *HS* health state

VAS preference values among all participants (*n* = 125) were ranked similarly to TTO preference values and generally consistent in magnitude. VAS preference values (mean [95% confidence interval]) ranked lowest to highest were refractory (0.15 [0.13–0.17]), relapse (0.21 [0.19–0.23]), transplant (0.29 [0.26–0.31]), treatment with chemotherapy (0.34 [0.32–0.37]), GvHD (0.38 [0.35–0.41]), consolidation (0.42 [0.40–0.45]), remission (0.53 [0.50–0.56]), and functionally cured (0.71 [0.68–0.73]).

Of the 120 participants in the main analysis population, 4 were identified for borderline response patterns (these patients were not included in the alternate analysis population). Of these 4 participants, 3 barely engaged in the TTO exercise (they did not trade off any year of life in 7 HSs, and traded only 1 year in the remaining HS), and 1 valued 7 HSs as equal to death (0) and the remaining HS as 0.1. Exclusion of these participants showed little impact on TTO estimates (4 HS mean preference values were 0.01 lower and 4 were unchanged; Table 3).

### Time trade-off preference values by gender and age

Analysis of preference values by gender revealed that women valued HSs slightly higher than men; however, significant differences in preference values were only observed between men and women for GvHD (*P* = 0.044) and remission (*P* = 0.053), which trended toward statistical significance (Table 4).

**Table 4:** Time Trade-Off Preference Values by Gender

Health State^a^	Men (*n* = 56)	Women (*n* = 64)	*P* Value^b^
HS-1 Treatment with chemotherapy	0.33 (0.47)	0.38 (0.36)	0.51
HS-2 Consolidation	0.44 (0.37)	0.48 (0.33)	0.50
HS-3 Transplant	0.25 (0.52)	0.31 (0.42)	0.54
HS-4 GvHD	0.35 (0.47)	0.50 (0.29)	0.04
HS-5 Remission	0.56 (0.31)	0.67 (0.25)	0.05
HS-6 Relapse	0.03 (0.59)	0.16 (0.46)	0.20
HS-7 Refractory	− 0.12 (0.52)	−0.11 (0.57)	0.88
HS-8 Functionally cured	0.75 (0.24)	0.76 (0.21)	0.88

*GvHD* graft-versus-host disease, *HS* health state

^a^Data are mean (standard deviation)

^b^Two-sample *t* test

The relationship between the preference valuations and age (analyzed as a continuous variable) was also explored. While all correlations showed lower preference values with increasing age (correlations were all negative), the correlation coefficients of age with TTO values were very low and did not reach statistical significance (*P* > 0.05). Spearman rank correlation coefficients were − 0.025 (*P* = 0.783) for treatment with chemotherapy, − 0.122 (*P* = 0.184) for consolidation, − 0.014 (*P* = 0.877) for transplant, − 0.109 (*P* = 0.236) for GvHD, − 0.090 (*P* = 0.326) for remission, − 0.071 (*P* = 0.443) for relapse, − 0.135 (*P* = 0.143) for refractory, and − 0.137 (*P* = 0.137) for functionally cured.

## Discussion

Long-term prognosis for patients with AML is poor. Additionally, HRQoL in patients with AML is significantly lower than the general population, with greater reductions observed in newly diagnosed patients [13, 14]. Therefore, understanding the impact of therapeutic modalities on HRQoL and societal preference values (or utility values) for HSs is important in assessing the benefits of treatments.

Additionally, preference values for HSs are used in cost-effectiveness analyses, a requirement by NICE for health technology appraisal in the UK [16]. This analysis defined 8 HSs for AML based on a literature review and discussions with practicing hematologists. The HSs reflected the experience of newly diagnosed adult patients with AML undergoing chemotherapy and hematopoietic stem cell transplantation. Valuation of HSs followed recommendations by NICE [16] and adopted a societal perspective and used a preferred choice-based preference elicitation method, TTO.

Overall, participants were co-operative, and the majority of participants had no difficulty answering the questions. Five of the participants did not sufficiently engage in the TTO exercise (ie, they were unwilling to trade years of life), a problem encountered in utility assessment [25], and their responses were removed from the TTO preference values calculation. Another 4 cases were also identified as being borderline in terms of their response patterns. Sensitivity analyses excluding participants with borderline responses did not affect final TTO valuation.

Preference values aligned with clinical perception, with poorer HSs rated lower by participants and better HSs rated higher. The order of ranking for HSs was consistently maintained in TTO and VAS valuations. “GvHD” and “consolidation therapy” valuations were comparable (0.43 and 0.46, respectively), indicating that participants view these states similarly. Comparison of the relative positions of GvHD versus consolidation therapy in female (0.50 vs 0.48, respectively) and male (0.35 vs 0.44, respectively) participants showed a reversal in position. “Relapse” (0.1) and “refractory” (− 0.11) were clearly differentiated by participants, with the latter considered worse than death.

Although preference values aligned with clinical perception for AML as a disease, they generally represented other acute leukemias as milder disease. Health state utility values derived from a preference elicitation study conducted in the UK for patients with relapsed/refractory acute lymphoblastic leukemia (R/R ALL) demonstrated higher score values compared with AML preference values obtained in this study [26]. Although progressive disease (0.3) was not an HS defined in this study, it is akin to the relapse HS (0.1) used herein and was valued higher in patients with ALL.

No preference values were rated worse than death for ALL. In contrast, refractory disease was valued as worse than death (− 0.1) for AML. The higher preference values for R/R ALL HSs compared with AML are surprising given that R/R ALL is also associated with poor prognosis. Overall survival for patients with ALL after relapse is < 25% at 1 year and decreases to < 10% at 5 years [27].

Furthermore, survival decreases with each successive line of salvage treatment, indicative of worse outcomes for patients with multiple relapses [28]. The 2 studies developed preference values for different hematological malignancies, albeit both acute leukemias; therefore, the results should be interpreted cautiously and may not be directly comparable. However, these results suggest that differences in descriptive terminology used to portray the patient’s experience with a disease and how severely disease symptoms are characterized may influence valuation of HSs.

Although the HSs in the present study described the general experience of a newly-diagnosed patient with AML, the perception of symptom severity of the HSs can vary greatly from patient to patient in clinical practice. An inherent limitation of this study is that these HSs are heterogeneous and the HS descriptions could potentially bias the ratings provided by the study participants. However, participants were presented with the most common experiences a patient with AML would encounter in each HS, and participants reported preference values that were consistent with clinical perception of HS severity.

A surprising finding in this study was the high rate of lifetime hematological disorders observed in participants’ health history; however, participants may have interpreted the hematological disorders as inclusive of nonmalignant conditions because the wording of the question was not specific to malignant hematological conditions. A potential strength of this study was that interviews were conducted one-on-one and administered by trained professionals skilled in face-to-face interviews. Interviewers were given intensive instruction for this study, and role playing sessions with complete mock interviews were implemented to ensure consistent quality of the interviews.

Moreover, language used in the HSs was written so that it could easily be understood by a layperson; cancer- or leukemia-specific terminology was avoided in HS descriptions and labels to prevent negative impact on valuation [29]. Lastly, a sampling procedure was put in place to ensure a fair representation of the population distribution across the country.

Application of condition-specific utility values and generic HRQoL measures, such as the EQ-5D, provide complementary information that can inform treatment decisions and resource allocation for AML. Both measures provide valuable information from different perspectives, but condition-specific utility values focus on specific symptoms- and treatment-related HSs, whereas EQ-5D scores focus on the patient’s health status from a functional, physical, and mental health perspective [18, 30]. Approximately 5% to 30% of patients with AML reported slight or moderate problems in the EQ-5D domains of anxiety, pain, problems with usual activities, or mobility [13].

Such a finding is consistent with the lower valuation of HSs in our study that are more likely to incur a higher frequency of anxiety, pain, or mobility issues, such as refractory or relapsed disease, transplantation, and treatment with chemotherapy. Congruence or lack thereof between HRQoL assessments and HS preference values may offer insight into which treatments are likely to provide the greatest benefit for patients, as well as provide useful information on cost-effectiveness.

## Conclusions

To our knowledge, this is the first study to report utility values for AML from the UK societal perspective. Although utility values were quite low for certain HSs, participants were able to distinguish differences in severity among AML HSs, and preference values were consistent with clinical perception of HS severity, particularly for the refractory and relapse HSs. HS preference values observed in this study may be useful in future evaluations of treatment benefit, including cost-effectiveness analyses and improved patient well-being.

## Abbreviations

ALL: Acute lymphoblastic leukemia
AML: Acute myeloid leukemia
BTD: Better than death
CI: Confidence interval
EQ-5D: EuroQol 5-Dimension Questionnaire
GvHD: Graft-vs-host disease
HRQoL: Health-related quality of life
HS: Health state
IRB: Institutional review board
NICE: National Institute of Health and Clinical Excellence
QALY: Quality-adjusted life-year
R/R ALL: Relapsed/refractory acute lymphoblastic leukemia
TTO: Time trade-off
VAS: Visual analog scale
WTD: Worse than death
